# A functional proteomics platform to reveal the sequence determinants of lysine methyltransferase substrate selectivity

**DOI:** 10.1126/sciadv.aav2623

**Published:** 2018-11-28

**Authors:** Evan M. Cornett, Bradley M. Dickson, Krzysztof Krajewski, Nicholas Spellmon, Andrew Umstead, Robert M. Vaughan, Kevin M. Shaw, Philip P. Versluis, Martis W. Cowles, Joseph Brunzelle, Zhe Yang, Irving E. Vega, Zu-Wen Sun, Scott B. Rothbart

**Affiliations:** 1 Center for Epigenetics, Van Andel Research Institute, Grand Rapids, MI 49503, USA.; 2 Department of Biochemistry and Biophysics, University of North Carolina at Chapel Hill, Chapel Hill, NC 27599, USA.; 3 Department of Microbiology, Immunology, and Biochemistry, Wayne State University School of Medicine, Detroit, MI 48201, USA.; 4 Department of Translational Science and Molecular Medicine and Integrated Mass Spectrometry Unit, College of Human Medicine, Michigan State University, Grand Rapids, MI 49503, USA.; 5 EpiCypher Inc., Research Triangle Park, NC 27709, USA.; 6 Advanced Photon Source, Argonne National Laboratory, Argonne, IL 60439, USA.

## Abstract

Lysine methylation is a key regulator of histone protein function. Beyond histones, few connections have been made to the enzymes responsible for the deposition of these posttranslational modifications. Here, we debut a high-throughput functional proteomics platform that maps the sequence determinants of lysine methyltransferase (KMT) substrate selectivity without a priori knowledge of a substrate or target proteome. We demonstrate the predictive power of this approach for identifying KMT substrates, generating scaffolds for inhibitor design, and predicting the impact of missense mutations on lysine methylation signaling. By comparing KMT selectivity profiles to available lysine methylome datasets, we reveal a disconnect between preferred KMT substrates and the ability to detect these motifs using standard mass spectrometry pipelines. Collectively, our studies validate the use of this platform for guiding the study of lysine methylation signaling and suggest that substantial gaps exist in proteome-wide curation of lysine methylomes.

## Teaser

Mapping lysine methyltransferase substrate selectivity reveals gaps in the proteome-wide curation of lysine methylomes.

## INTRODUCTION

Reversible protein posttranslational modifications (PTMs) (e.g., acetylation, ubiquitination, phosphorylation, and methylation) are key regulators of protein activity, stability, subcellular localization, and molecular interactions (*1*–*4*). While lysine methylation was found more than a half century ago (*5*), the study of protein signaling through this PTM did not intensify until the early 2000s when histone lysine methylation was connected to transcriptional regulation (*6*). Since then, substantial effort has been devoted to the study of lysine methylation signaling, primarily in the context of histone proteins.

Methylation on the ε-amine of the lysine side chain is catalyzed by a family of approximately 60 lysine methyltransferase (KMT) enzymes (*4*). While numerous KMTs are bona fide histone methyltransferases, many have little or no activity toward histone proteins. The number of identified KMTs with nonhistone substrates is steadily growing (*7*) but is being outpaced by the discovery of methylated proteins by mass spectrometry (MS). More than 6000 unique sites of lysine methylation on more than 3000 unique human proteins have been identified by MS (*8*), but very few of these identified sites have been linked to a KMT. The inability to connect KMTs to their preferred substrates is a barrier to fully appreciating the biological roles of lysine methylation.

Here, we report on the development of a functional proteomics platform to enable rapid mapping of KMT substrate selectivity without a priori knowledge of a substrate. Our approach uses a lysine-oriented peptide library (K-OPL) to generate a KMT substrate selectivity profile (±3 amino acids from a fixed central lysine) for any KMT. Sequence maps are used to rank all lysine-centered motifs in any proteome of interest by the likelihood of its use as a substrate. Variations of this positional scanning technology have been used to map the substrate selectivity of kinases and arginine methyltransferases (*9*, *10*).

To validate the K-OPL approach for KMTs, we confirm and expand upon the known substrate motifs of G9a (EHMT2/KMT1C), SET7/9 (SETD7/KMT7), and SMYD2 (KMT3C). We further demonstrate how K-OPL data can be used to reveal novel and kinetically distinct substrates for these enzymes, to discover inhibitor scaffolds, and to identify cancer-associated missense mutations that may modulate lysine methylation signaling by altering, removing, or creating new KMT substrates. Notably, we discover that the substrates most preferred by the enzymes characterized in this study are difficult to detect using standard bottom-up MS proteomics pipelines. The implications of these observations are important for the future study of lysine methylation signaling and suggest that the current compendium of lysine methylation sites curated from MS proteomics datasets may be substantially underrepresented. Overall, this study validates the use of the K-OPL platform for mapping KMT substrate selectivity and demonstrates ways in which data generated with this platform can guide the biochemical and biological study of lysine methylation signaling.

## RESULTS

### Development of a KMT screening platform that queries K-OPL

**Fig. 1:**
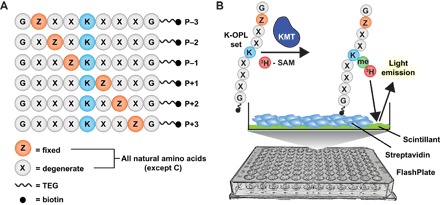
K-OPL platform for mapping KMT substrate selectivity. (**A**) Composition and design of the K-OPL. (**B**) Cartoon depiction of the SPA developed for screening the activities of KMTs with K-OPL. TEG, triethylene glycol; SAM, *S*-adenosylmethionine.

The K-OPL used in this study consisted of approximately 47 million unique peptides. Each peptide was nine amino acids long, oriented around a lysine residue at the fifth position (Fig. 1A). Fixed N- and C-terminal glycine residues were included for spacing, and each peptide was C-terminally functionalized with triethylene glycol–biotin to enable surface immobilization. Peptides were divided into 114 sets. Each set was made by fixing an additional amino acid ±3 from the central lysine and varying the other five positions uniformly among 19 synthetically tractable natural amino acids (excluding cysteine). On the basis of structural information for numerous KMTs, lysine demethylases (KDMs), and readers bound to histone peptides, we rationalized that three residues flanking either side of the central lysine provide a sufficient footprint for target engagement while also minimizing the total number of sets required to capture the selectivity of a KMT.

To measure enzyme activity, we developed a scintillation proximity assay (SPA), which quantified the transfer of tritium-labeled methyl groups from the cofactor and methyl donor *S*-adenosylmethionine (SAM) to each K-OPL set (Fig. 1B). SPAs permit sensitive analysis of enzymatic activity and are advantageous when the reaction product is unknown. This platform enabled us to rapidly determine a KMT’s preference for any amino acid (except cysteine) at any position ± three positions from a target lysine.

### Generation of KMT substrate selectivity profiles with K-OPL

**Fig. 2:**
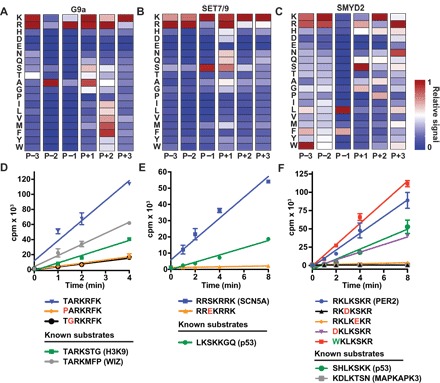
K-OPL reveals the substrate selectivity of G9a, SET7/9, and SMYD2. K-OPL substrate selectivity profiles for G9a (**A**), SET7/9 (**B**), and SMYD2 (**C**). Mean results of two independent K-OPL SPA screens for each enzyme are reported as position-normalized heat maps (see fig. S1 for global normalized heat maps and raw K-OPL data). The color code is proportional to the creation of enzyme product, where red (1) is most active and blue (0) is least active. Rows show the identity of each fixed residue, and columns show the position within the sequence. Initial rate measurements with peptides corresponding to known and newly identified substrates for G9a (**D**), SET7/9 (**E**), or SMYD2 (**F**). cpm, counts per minute. Point mutations predicted to decrease or increase the rate of methylation are indicated in red or green, respectively. Data points are shown as the mean of three independent measurements, and error is presented as ±SEM. For some data points, error bars are masked by the symbol weight.

To test whether the K-OPL platform could derive the sequence determinants of KMT substrate selectivity, we profiled G9a, SET7/9, and SMYD2; each of which have been reported to methylate distinct histone and nonhistone substrates. The substrate selectivity profiles obtained using K-OPL were unique for each of these three KMTs (Fig. 2, A to C). Each profile was consistent with the amino acid composition of previously identified substrates, and new selectivity information was also found.

G9a catalyzes monomethylation and dimethylation of lysine 9 on histone H3 (H3K9me1/me2) (*11*) and has several reported nonhistone substrates, including K1162me3 on WIZ, K135me3 on CDYL1, and K654me2 on ACINUS (*12*). The reported G9a substrate motif, XAR-K-SXX, corresponds to the sequence surrounding H3K9 and many of its nonhistone substrates. The K-OPL sets that correspond to this sequence (P−2 A, P−1 R, and P+1 S) were among the most methylated by G9a (Fig. 2A and fig. S1, A and B). Of all the sets with a fixed amino acid at the P−3 position, the set with fixed threonine (which corresponds to the sequence context surrounding H3K9) was the third most used. The G9a substrate selectivity profile derived by K-OPL also revealed preference for amino acids at the P+2 and P+3 positions, which did not match the sequence surrounding H3K9. For example, the sets containing hydrophobic amino acids (I, L, V, M, and F) at the P+2 position were good substrates (Fig. 2A), a finding consistent with the previous analysis of G9a substrate preference (*12*). Preferential substitutions to the H3K9 sequence at the P+1, P+2, and P+3 positions (TARKRFK) created a more efficient substrate than H3K9 or WIZ (Fig. 2D). In addition, substitutions in the TARKRFK sequence to amino acids predicted by the K-OPL selectivity profile to be less favorable (threonine to proline in the P−3 position or alanine to glycine in the P−2 position) resulted in substrates with severely reduced methylation rates (Fig. 2D).

SET7/9 was first reported to monomethylate H3K4 (H3K4me1) (*13*) but was later shown to target K189me1 on TAF10, K372me1 on p53, and numerous other proteins (*14*). Based on validated substrates and structural studies, the consensus SET7/9 motif is X[KR][STA]-K-pXX, where p denotes a polar amino acid. Like G9a, the K-OPL substrate selectivity profile for SET7/9 was consistent with the reported motif (Fig. 2B and fig. S1, C and D). The P−1 serine K-OPL set was the most used at this position. In the P+2 position, several K-OPL sets with polar amino acids were well used, including lysine, arginine, histidine, asparagine, glutamine, and serine. The SET7/9 K-OPL footprint suggested that H3K4 is not an optimal substrate. The optimal SET7/9 substrate predicted by K-OPL screening was RRSKRRK, a sequence that maps to K477 of SCN5A (the α subunit of the cardiac sodium channel). SCN5A K477 was methylated at a faster rate than p53 K372 (Fig. 2E). As predicted by the K-OPL substrate selectivity profile, substitution of the P−1 serine in RRSKRRK for glutamic acid resulted in loss of any detectable methylation.

SMYD2 has been reported to monomethylate or dimethylate histone H3 (H3K4me1 and H3K36me2) (*15*, *16*), p53 (K370me1) (*17*), RB (K860me1) (*18*), MAPKAPK3 (K355me1) (*19*), and numerous other proteins (*20*). It has been reported that SMYD2 prefers a XX[LFM]-K-SXX motif, and all K-OPL sets corresponding to this reported motif were among the most methylated by SMYD2 (Fig. 2C and fig. S1, E and F). Despite initial reports that SMYD2 methylates H3K4 and H3K36, K-OPL analysis suggests that both would be poor substrates. In vitro methyltransferase assays using recombinant human mononucleosomes confirmed that unlike G9a, recombinant human nucleosomes are poor SMYD2 substrates compared to a pool of all K-OPL sets (fig. S2A).

To validate the SMYD2 K-OPL screen, a series of peptides were synthesized on the basis of the optimal substrate, WKLKSKR. This exact sequence is not found in any human protein. However, substitution of the P−3 tryptophan with arginine corresponds to lysine 798 of PER2 (RKLKSKR), a transcriptional repressor and core component of the circadian clock. As predicted by the K-OPL substrate selectivity profile, a peptide corresponding to PER2 K798 was a more efficient substrate than peptides corresponding to previously reported SMYD2 substrates p53 K370 and MAPKAPK3 K355 (Fig. 2F). Substitution of aspartic acid in the P−1 position, glutamic acid in the P+1 position, or aspartic acid in the P−3 position reduced the rate of methylation by SMYD2, while substitution to tryptophan at the P−3 position increased the rate of methylation (Fig. 2F).

To further validate specific KMT peptide substrates revealed by K-OPL, we performed comparative rate measurements for the best SMYD2, G9a, and SET7/9 substrates with each enzyme (fig. S2B). These results demonstrate that sequence compositions revealed by K-OPL screening are highly enzyme specific.

**Fig. 3:**
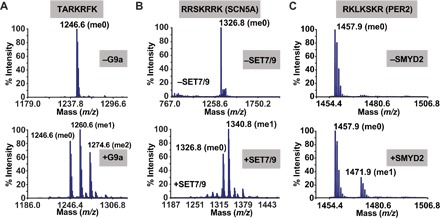
MS analysis of methylation products. The products from reactions of G9a (**A**), SET7/9 (**B**), and SMYD2 (**C**) with their corresponding peptide substrates were analyzed by MS. Mass spectra are shown in the absence (top) or presence (bottom) of enzyme treatment, as indicated.

All three KMTs screened in this study preferred basic residues near the central lysine. The resulting substrates predicted by the K-OPL selectivity map often contained more than one lysine residue, including the optimal substrates tested for SMYD2 (RKLKSKR), G9a (TARKRFK), and SET7/9 (RRSKRRK). To rule out the possibility that multiple methylation events on single peptides contributed to the increased substrate utilization seen in our assays, we performed MS analysis on the products of these methylation reactions. G9a methylation of TARKRFK resulted in both monomethyl and dimethyl products (Fig. 3A). Tandem MS (MS/MS) analysis of both products indicated that only the central lysine of this substrate was methylated (fig. S3, A and B). For SET7/9 and SMYD2, MS analysis detected a mass shift consistent with the addition of a single methyl group (Fig. 3, B and C), and MS/MS confirmed that only the central lysine was monomethylated (fig. S3, C and D).

Collectively, K-OPL analysis accurately identified and extended known sequence motifs for G9a, SET7/9, and SMYD2. In addition, K-OPL substrate selectivity profiles accurately predicted how changes in the substrate sequence negatively or positively affected its rate of methylation. These data validate the use of K-OPL for profiling KMT substrate selectivity and provide the first high-resolution maps of the amino acid preference for G9a, SET7/9, and SMYD2. Notably, these results suggest that the most studied substrates for these three enzymes are not the most robust.

### Structural and kinetic analysis of SMYD2-substrate interactions

We focused downstream efforts on SMYD2, in part, because its profile revealed that its most well-appreciated substrate, p53 K370, contains several nonoptimal residues. In addition, small-molecule inhibitors for a variety of clinical indications are currently under development for SMYD2 (*21*). These therapeutic efforts are primarily based on the biological connection that SMYD2 methylates the tumor suppressor protein p53, leading to inhibition of p53 function (*17*). The knowledge of all SMYD2 targets will be important for understanding the clinical outcomes of SMYD2 inhibition.

**Fig. 4:**
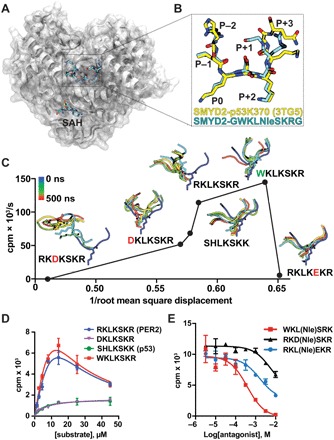
Structural and kinetic analysis of SMYD2. (**A**) Hybrid ribbon-surface representation of SMYD2 (white) bound to SAH and GWKLNleSKRG (Nle, norleucine) (blue sticks). Costructure has been deposited in the Protein Data Bank (PDB) as PDB: 6MON. (**B**) Overlay of peptide substrates from SMYD2-GWKLNleSKRG (PDB: 6MON) and SMYD2-p53K370 (PDB: 3TG5) structures. (**C**) Scatterplot comparing the relationship between the methylation rate calculated from Fig. 2F and the dynamics of substrate coordination by SMYD2. Root mean square displacement (RMSD) of the Cα atoms of the indicated peptides was calculated from 500-ns whole-atom MD simulations. Peptide orientations at several time points over the course of the MD simulations are shown, indicated by color with the corresponding color scale (top left). (**D**) Kinetic analysis of SMYD2 methylation of p53, PER2, and PER2 derivative substrates. Data points are the mean of three independent measurements, and error is presented as ±SEM. PER2 and WKLKSKR were fit to a substrate inhibition kinetic model. DKLKSKR and SHLKSKK were fit to a standard Michaelis-Menten (MM) model. (**E**) IC_50_ (median inhibitory concentration) measurements of Nle peptide inhibitors of SMYD2 using PER2 as a substrate. Data points are the mean of three independent measurements, and error is presented as ±SEM.

To determine the molecular basis for SMYD2 substrate selectivity, we first solved a 2.7-Å x-ray crystal structure of SMYD2 bound to *S*-adenosylhomocysteine (SAH) and a peptide with the sequence GWKL-Nle-SKRG (Fig. 4, A and B; fig. S4, A and B; and table S1). This peptide corresponded to the optimal K-OPL–derived SMYD2 substrate with the central lysine substituted for norleucine (Nle), a methyl-lysine mimic that stabilizes KMT-substrate complexes (*22*). The P−3 tryptophan was not resolved in the structure, suggesting conformational flexibility in this position (fig. S4A). Unexpectedly, the peptide conformation was nearly identical to previous SMYD2-SAH-peptide structures (Fig. 4B and fig. S4B) (*23*–*25*), offering little insight as to why PER2 or WKLKSKR are better substrates than p53.

Next, we collected a series of 0.5-μs molecular dynamic (MD) trajectories (3 μs in total) of SMYD2 in complex with various substrates (Fig. 4C). As a starting template, we used the structure of SMYD2 bound to a p53 peptide (PDB: 3TG5), which resolved residues between the P−2 and P+3 positions relative to p53 K370. The unresolved amino acid in the P−3 position was modeled, and all-atom MD simulations of this structure in complex with the peptides used for in vitro KMT assays allowed us to evaluate the dynamics of SMYD2-substrate interactions. The RMSD of the Cα atoms in the peptide backbone throughout the simulation (Fig. 4C, *x* axis) provided a proxy for peptide stability, or off-rate, because simulations began in the bound state. Integration of this metric with in vitro KMT reaction rate measurements (Fig. 2F) led to the hypothesis that coordination of SMYD2 substrates and catalytic turnover are related by a quasi-concave function (Fig. 4C). In this model, loosely coordinated peptides (i.e., RKDKSKR) are poor substrates because the enzyme-substrate interaction is too weak to organize the substrate for catalysis. Tightly coordinated peptides (RKLKEKR) are also poor substrates because of their slow off-rates, leading to inefficient substrate turnover. Optimal substrates (KLKSKR and WKLKSKR) are organized to allow for both efficient catalysis and rapid turnover.

The MD simulations also identified unique interactions for the SMYD2 substrates PER2 and WKLKSKR. The PER2 peptide quickly moved to an alternate conformation to make stabilizing contacts at the P−3 position. In this conformation, the P−3 arginine formed a salt bridge with D151 of SMYD2 (fig. S4C). The P−3 tryptophan of the WKLKSKR peptide settled in a hydrophobic pocket (fig. S4D) near the helix that contained D151. This is the same pocket occupied by AZ506, a recently found small-molecule inhibitor of SMYD2 (fig. S4E) (*26*). Overall, the MD simulations revealed unique interactions for both PER2 and WKLKSKR, which likely contribute to the faster methylation rates observed for these peptides.

Next, we performed kinetic analysis to further investigate what makes PER2 and WKLKSKR better SMYD2 substrates than p53. SMYD2 methylation of p53 adhered to classical MM kinetics (Fig. 4D). However, SMYD2 methylation of PER2 and WKLKSKR had a strikingly different kinetic profile, consistent with substrate inhibition. Increased concentrations of PER2 or WKLKSKR resulted in decreased rates of methylation.

Because the MD simulations revealed unique P−3 conformations for the non-MM substrates (Fig. 4C and fig. S4, C and D), we next questioned whether these interactions contribute to the observed substrate inhibition kinetics. In MD simulations, substitution of an aspartic acid in the P−3 position prevented the peptide from interacting with SMYD2 in the same manner as PER2 or WKLKSKR (Fig. 4C and fig. S4, C and D), and SMYD2 methylation of DKLKSKR followed a classical MM model (Fig. 4D). Together, these observations suggest that the amino acid composition of the P−3 position modulates the conformation of SMYD2 substrates and the kinetics of SMYD2.

### K-OPL as a tool for discovering scaffolds for rational KMT inhibitor design

Replacement of a substrate’s target lysine with Nle has been previously used as an inhibitory strategy for KMTs (*22*). As a proof of concept for K-OPL–guided discovery of KMT inhibitors, we synthesized Nle-containing peptides based on the optimal SMYD2 substrate and found that the Nle derivative of WKLKSKR inhibited SMYD2 methylation of PER2 (Fig. 4E). RKDKSKR and RKLKEKR Nle derivatives were less effective, as predicted by K-OPL analysis. However, although RKLKEKR and RKDKSKR were equally poor SMYD2 substrates (Fig. 2F), RKLNleEKR was a more efficient competitive inhibitor than RKDNleSKR (Fig. 4E). This result was consistent with the MD simulation that showed that RKLKEKR formed a more stable complex with SMYD2 (Fig. 4C). Overall, these results suggest that optimal substrates identified by K-OPL screens can serve as scaffolds for further optimization toward more potent inhibitors.

### Using K-OPL to identify new SMYD2 substrates

To identify new KMT substrates, we developed a lowest bin (LoB) scoring function based on K-OPL selectivity profiles to rank lysine-centered 7-mer sequences from annotated proteomes. LoB scores are equal to the raw signal from the lowest K-OPL set used to construct an entire 7-mer sequence, e.g., consider PER2 K798 (RKLKSKR). The score for this sequence is assigned by the arginine in the P−3 position, as this set has the lowest signal in this sequence (fig. S1, E and F). The LoB score was purposely designed to minimize false positives and contains no positional weighting common to other motif scoring functions (*27*, *28*).

**Fig. 5:**
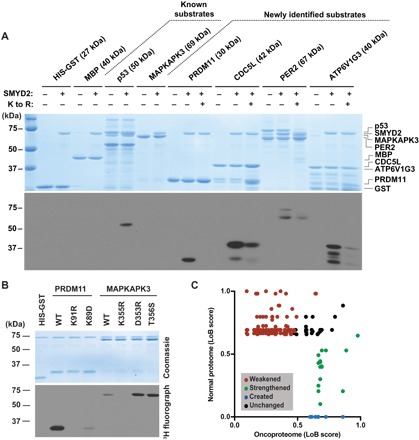
Novel SMYD2 substrates identified with K-OPL. (**A**) Representative in vitro SMYD2 methyltransferase assay with known and predicted protein substrates. K to R refers to a missense mutation (lysine to arginine) introduced at the target lysine. Coomassie-stained gel is shown on top in blue, and ^3^H fluorography is shown on the bottom. (**B**) Representative in vitro SMYD2 methyltransferase assay with mutant forms of PRDM11 and MAPKAPK3 substrates predicted to decrease or increase substrate efficiency, respectively. WT, wild-type. (**C**) Scatterplot of the LoB score for SMYD2 methylation motifs that are created (blue), weakened (red), strengthened (green), or unchanged (black) by missense mutations found in primary human cancer sequencing data.

A candidate list of six proteins (PER2, PRDM11, CDC5L, GDAP1, ZPK, and ATP6V1G3) from the top 50 LoB-scored sequences (table S2) was selected for in vitro validation as SMYD2 substrates. All six proteins were methylated by SMYD2 (Fig. 5A and fig. S5A). ZPK and GDAP1 were poor SMYD2 substrates (fig. S5A). Available structural data for ZPK showed that the target lysine is in a structured helix that likely prohibited methylation by SMYD2 (fig. S5B), but no structural data were available to rationalize why GDAP1 is not a more robust SMYD2 substrate. To determine whether the lysine predicted by the K-OPL screen was methylated, we generated protein substrates with the target lysine substituted to arginine (K to R). All K to R mutant substrates had reduced methylation (Fig. 5A and fig. S5A). For CDC5L, ATP6V1G3, and PER2, the K to R mutation did not completely abolish methylation, suggesting that additional residues are also being methylated. Consistent with rate measurements for the MAPKAPK3 K355 peptide (Fig. 2F), methylation of full-length recombinant MAPKAPK3 was weaker than most of the newly identified SMYD2 substrates (Fig. 5A), requiring a much longer exposure to detect methylation of this protein (fig. S5A).

Overall, SMYD2 methylated four of the newly identified substrates (PER2, PRDM11, CDC5L, and ATP6V1G3) at least as efficiently as the known substrate, p53. These results validate the use of K-OPL selectivity profiles to identify new KMT substrates.

### K-OPL analysis predicts the impact of missense mutations on substrate usage

Missense mutations have been shown to alter kinase signaling networks, including mutations that cause amino acid substitutions in proximity to the modified residue (*29*). We sought to determine whether K-OPL selectivity profiles could predict the impact of missense mutations on a KMT substrate at the protein level. Guided by the SMYD2 K-OPL profile, we generated a PRDM11 mutant (K89D), predicted to render this robust SMYD2 substrate deficient. In an in vitro KMT assay, PRDM11 K89D methylation was reduced compared to the wild-type PRDM11 (Fig. 5B). In addition, mutations predicted to enhance methylation of the weak SMYD2 substrate MAPKAPK3 (D353R and T356S) improved methylation of this protein (Fig. 5B). These results show that K-OPL–derived selectivity profiles accurately predict how single amino acid changes near the target lysine can significantly affect the efficiency of a KMT substrate at the protein level.

We next turned our attention to predicting how reported missense mutations in primary human cancer sequencing datasets might rewire lysine methylation signaling networks because of substitutions within SMYD2 substrate motifs. To do so, we catalogued and analyzed K-centric 7-mer amino acid sequences on a proteome-wide scale. Comparison of the LoB scores for SMYD2 targets in the normal proteome (UniProt) with the oncoproteome (COSMIC) (*30*) resulted in the identification of four classes of missense mutations that may affect SMYD2 lysine methylation signaling. The four classes include mutations that (i) weakened, (ii) strengthened, (iii) created, or (iv) had no effect on a target of methylation (Fig. 5C and table S3). These results demonstrate the utility of K-OPL datasets for prioritizing the study of cancer-associated missense mutations based on their predicted functional relationship with lysine methylation signaling. Furthermore, these studies suggest that the lysine methylome of an individual cancer cell may be altered due to missense mutations.

### K-OPL analysis reveals gaps in the lysine methylome

**Fig. 6:**
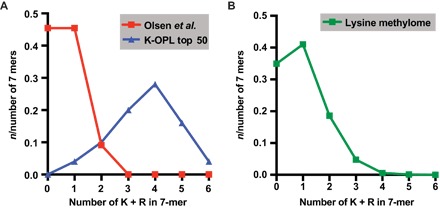
K-OPL analysis reveals a gap in MS-based lysine methylation datasets. (**A**) Comparison of the arginine and lysine content between the top 50 K-OPL–predicted SMYD2 substrates (blue) and the 35 substrates identified using MS (red) (Olsen *et al*.). (**B**) Lysine and arginine content in the entire lysine methylome as curated by PhosphoSitePlus (*8*).

A recent study used MS to identify 35 proteins with monomethylation sites that consistently decreased upon loss of SMYD2 activity (*20*). Surprisingly, these 35 proteins fall well outside our top 50 LoB-scored substrates (table S2). The physical properties of MS-identified substrates are distinct from the top 50 LoB-scored substrates. Most of the top 50 K-OPL–predicted substrates are enriched with lysine and arginine residues, whereas nearly all of the top 35 substrates from the Olsen *et al*. study (*20*) are depleted of these amino acids (Fig. 6A). The density of lysine and arginine positively correlates with hydrophilicity and is a proxy for the number of trypsin cut sites, all confounding the detection of these substrate motifs by MS. Thus, either the proteins that K-OPL identifies as ideal substrates are never methylated in cells or they are undetectable using standard bottom-up MS pipelines.

To test the latter hypothesis, we performed bottom-up MS analysis of recombinant PER2. Despite achieving more than 70% sequence coverage of the recombinant protein (fig. S6, A and B), we were unable to detect peptides corresponding to the region encompassing the newly identified K798 substrate for SMYD2. This suggests that even if PER2 or other basic motifs predicted by K-OPL analysis were methylated in cells, they would not be detected by MS.

We note that in addition to SMYD2, SET7/9 and G9a also prefer lysine- and arginine-rich substrates (Fig. 2). While this is a small sampling of KMT activities, analysis of the lysine and arginine content of human lysine methylation proteomics data curated in PhosphoSitePlus (*8*) revealed that the bias seen in the Olsen *et al*. study is reflective of the annotated lysine methylome (Fig. 6B). While it is well appreciated that lysine- and arginine-rich sequences are challenging to detect by MS (*31*), K-OPL analysis demonstrates that the enzymes studied here have a strong bias for substrates with the exact sequence compositions that escape MS detection. This key discovery suggests that the current compendium of lysine methylation sites curated from MS proteomics datasets is incomplete.

## DISCUSSION

Our study establishes K-OPL as a functional proteomics platform for mapping KMT substrate selectivity by quantitatively measuring amino acid preference ± three positions from the target lysine. In addition to the identification of new substrates, K-OPL can be used as a tool for discovering new KMT inhibitor scaffolds. K-OPL analysis of SMYD2 substrate selectivity revealed that the optimal substrate is not found in the human proteome (WKLKSKR) due to the tryptophan in the P−3 position. A Nle derivative of the optimal substrate with no further modification was a modest inhibitor of SMYD2 activity. MD simulations showed that the tryptophan settles in a hydrophobic pocket near the SMYD2 active site, and a recently found small-molecule inhibitor of SMYD2 contains a bulky aromatic group that also binds in this same pocket. Thus, the K-OPL screening platform identified a SMYD2-substrate interaction that would never have been found through the analysis of previously identified SMYD2 substrates. Use of K-OPL analysis to generate scaffolds for inhibitor development is an exciting future application, especially for KMTs with no known inhibitors.

In addition, the K-OPL platform can be applied to understand the impact of missense mutations on lysine methylation signaling. Our analysis revealed that missense mutations in human cancer can have a substantial impact on the lysine methylation signaling network for SMYD2. Missense mutations that strengthen, weaken, create, or destroy new SMYD2 substrates are abundant in human cancer cells. Already, this limited analysis motivates future work mapping how the lysine methylome changes as a consequence of missense mutations. As K-OPL profiling of additional KMTs is completed, this integrative analysis will become increasingly useful for formulating hypotheses on the role of lysine methylation in human disease.

K-OPL profiling of SMYD2 enabled the identification of new substrates. Identifying KMT substrates is a major challenge, and previously reported approaches have limitations that the K-OPL platform circumvents. For example, qualitative tolerance assays have been used to determine the impact of individual amino acids on a known KMT substrate and have been used successfully to identify new targets for G9a, SET7/9, and other KMTs (*12*, *32*). The K-OPL platform does not require any known substrate, which may help identify substrates of orphan KMTs, such as members of the enigmatic PR domain–containing family. We note that the K-OPL screening approach does not consider some significant contributions to substrate selectivity that may be present in a cell, such as complex membership, expression levels, tissue specificity, and subcellular localization. Careful consideration of these additional contributions to substrate selectivity can be used to further prioritize candidate substrates.

In addition to tolerance assays, new KMT substrates have been identified using MS analysis coupled with genetic or chemical genetic approaches. MS-based approaches suffer from two technical limitations. First, pan–methyl-lysine affinity reagents, required for enrichment before MS analysis, often contain a sequence bias, masking some of the lysine methylome (*10*, *33*). The K-OPL approach does not require an affinity enrichment step. Second, MS-based proteomics pipelines analyze peptides from primarily trypsin-digested cell lysates. A recent analysis of proteomics data deposited in the Global Proteome Machine Database (GPBdb) showed that 96% of all data were derived from trypsin-digested samples (*31*). All three of the KMTs screened in this study prefer arginine and lysine residues surrounding the substrate lysine. Tryptic digestion of the optimal sequence motifs produces peptides that are not detected by MS. Our analysis of a recent MS-based study of SMYD2 (*20*) demonstrates that the identified substrates are lysine and arginine deficient. Furthermore, we could not detect PER2 K798 by MS despite excellent sequence coverage, exemplifying the difficulty to detect lysine- and arginine-rich sequences by MS. K-OPL analysis of G9a, SET7/9, and SMYD2 reported in this study, and analysis of reported nonhistone substrates for other KMTs (fig. S6C), suggest that the annotated human lysine methylome is likely incomplete due to the basic sequence composition of the most preferred methylation motifs for these enzymes. This study highlights the need for developing new MS-based methods to detect lysine methylation in sequence compositions that are preferentially modified by KMTs but are undetectable by standard bottom-up MS pipelines.

Our knowledge of the enzymes responsible for the addition of nonhistone lysine methylation is lacking. Comprehensive mapping of KMT substrates is a critical step toward the goal of understanding the many biological roles of lysine methylation. Progress toward this important and challenging goal will require the development of new technologies and techniques to study lysine methylation. This report validates the use of K-OPL as a robust substrate selectivity screening platform that can now be used to further guide the study of lysine methylation signaling.

## MATERIALS AND METHODS

### Recombinant protein production

SET7/9 was purchased from New England BioLabs (catalog no. M0223). G9a (amino acids 913 to 1210) was produced as a 6XHis N-terminal fusion, and SMYD2 (full length) was expressed as a N-terminal glutathione *S*-transferase (GST) fusion. MAPKAPK3 (amino acids 1 to 520) (catalog no. 131688) and GDAP1 (full length) (catalog no. 162725) were obtained from Abcam. PRDM11 (amino acids 79 to 314) (Addgene plasmid no. 32858) and full-length human TP53 (Addgene plasmid no. 24859) were gifts from C. Arrowsmith. PER2 (amino acids 691 to 900) was subcloned into a modified pQE vector as an N-terminal maltose-binding protein (MBP)–His fusion. MAPKAPK3 (full length), ATP6V1G3 (full length), and CDC5L (amino acids 150 to 280) were subcloned into pGEX-6P2 (GE Healthcare) as N-terminal GST fusions. Point mutations were generated by QuikChange Site-Directed Mutagenesis (Stratagene). All expression constructs were transformed into *Escherichia* *coli* BL21(DE3) and grown in LB media (Caisson) at 37°C. When the OD_600_ (optical density at 600 nm) reached 0.6 to 0.8, the temperature was lowered to 16°C, isopropyl-β-d-thiogalactopyranoside was added (0.5 mM), and incubation was continued overnight with shaking. Bacteria were harvested by centrifugation and either frozen at −80°C or used immediately. Protein was purified with either glutathione agarose (GE Healthcare) or TALON resin (Clontech) according to the manufacturer’s protocol.

### K-OPL synthesis

All 114 peptide sets were synthesized on a PTI Symphony peptide synthesizer using Fmoc chemistry. The sets were synthesized on Biotin-PEG NovaTag resin (10 μmol per set; MillporeSigma no. 855055) using a single 70-min coupling with 12-fold excess of coupling mixture (amino acids/HATU/3-eq *N*-methylmorpholine) and 2× 10-min deprotection with 20% piperidine in *N*,*N*′-dimethylformamide (DMF). Degenerate positions were synthesized using a mixture of 19 Fmoc-protected l-amino acids (cysteine was excluded) at molar ratios consistent with coupling efficiency, as previously described. After final Fmoc deprotection, resins were washed (3× DMF, 3× dichloromethane, and 3× methanol) and left overnight under high vacuum. Resins were mixed 2 hours with 0.5 ml of cleavage mixture (92.5% trifluoroacetic acid, 2.5% H_2_O, 2.5% triisopropylsilane, and 2.5% 1,2-ethanedithiol) and precipitated with cold diethyl ether. Precipitates were washed with diethyl ether and separated by centrifugation. The washing procedure was repeated five times. After separation, the precipitates were air dried for 5 min, dissolved in 1 ml of 50% acetonitrile, frozen at −80°C, and dried on a speed-vac overnight. To assess the quality of the libraries, matrix-assisted laser desorption/ionization–time-of-flight (MALDI-TOF) MS spectra were collected for each library using SCIEX TOF/TOF 5800 MALDI MS spectrometer and compared with theoretical mass distributions (analytical data are available upon request).

### SPA for KMTs

Reactions (10 μl) containing 1 μg of KMT, 1 μg of a K-OPL set, and 1 μCi of ^3^H-SAM (PerkinElmer) in KMT reaction buffer [50 mM tris (pH 8.8), 5 mM MgCl_2_, and 4 mM dithiothreitol] were incubated for 1 hour at room temperature. Reactions were stopped by adding trifluoracetic acid to a final concentration of 0.5%, neutralized by diluting with 135 μl of 50 mM NaHCO_3_, and transferred to streptavidin-coated FlashPlates (PerkinElmer). Plates were incubated for 15 min, sealed, and counted in a MicroBeta2 liquid scintillation counter (PerkinElmer) for 1 min per sample. Initial rate measure and kinetic analysis were performed using the same procedure with 200 nM SMYD2, 50 μM SAM (5:1 cold/hot ratio), and 80 μM substrate (initial rate measurements) or as indicated (kinetic analysis). IC_50_ measurements were performed using the same conditions with 5 μM PER2 peptide as a substrate.

### In vitro KMT reactions

Reactions (10 μl) containing 1 μg of KMT, 1 μg of the indicated substrates, and 1 μCi of ^3^H-SAM (PerkinElmer) in KMT reaction buffer were incubated for 1 hour at room temperature. Reactions were quenched by the addition of SDS loading buffer and resolved by SDS–polyacrylamide gel electrophoresis. Following the detection of total protein by Coomassie staining, gels were treated with EN^3^HANCE (PerkinElmer) and dried, and methylated proteins were detected by autoradiography.

### MALDI-TOF-MS analysis of KMT reactions

For MS experiments, 200 nM KMT, 80 μM peptide, and 50 μM SAM were incubated in KMT reaction buffer for 1 hour at room temperature. Reactions were quenched with 0.5% trifluoroacetic acid and analyzed by MS. KMT-reacted samples were deposited on a MALDI target plate (4 μl per spot) and mixed with 1 μl of matrix solution (α-cyano-4-hydroxycinnamic acid in 50% acetonitrile). MALDI-TOF-MS and MS/MS (positive ion mode at 1 kV) spectra were collected using SCIEX TOF/TOF 5800 MALDI MS spectrometer. The peptide fragmentation modeling and peak assignments were done using the Peptide Sequence Fragmentation Modeling tool (https://omics.pnl.gov/software/molecular-weight-calculator).

### LC-MS/MS analysis of PER2

Recombinant MBP-tagged PER2 was expressed and purified as described above. Three micrograms of MBP-PER2 was buffer exchanged into 25 mM ammonium bicarbonate (pH 8.0). The sample was dried using a speed-vac, reconstituted in 25 mM ammonium bicarbonate (pH 8.0):50% acetonitrile, and incubated at 37°C for 1 hour. Trypsin, Arg-C, or Asp-N (500 ng each; Promega) was added, and samples were digested overnight at 37°C. The resulting peptides were dried and reconstituted in 25 mM ammonium bicarbonate:5% acetonitrile. Samples were loaded onto a C18 column (2-μm particles, 25-cm by 75-μm inner diameter) and eluted using a 2-hour acetonitrile gradient into a Q Exactive HF-X mass spectrometer, equipped with a nanospray source (flow at 350 nl/min). Full MS resolutions were set to 60,000 at 200 *m/z* (mass/charge ratio), full MS automatic gain control (AGC) target was 3 × 10^6^, and mass range was set to 300 to 1400. AGC target value for fragment spectra was set at 1 × 10^5^, intensity threshold was set at 2 × 10^5^, and isolation width was at 1.3 *m/z*. Normalized collision energy was set at 28% (*34*). The mass spectra from each sample were searched against the UniProt human database and a custom database containing the sequence of our MBP-PER2 construct using Proteome Discoverer (version 2.2). Precursor mass tolerance was set to 10 parts per million, fragment mass tolerance was set at 0.02 Da, Delta Cn of 0.05, false discovery rate of 0.01, minimum peptide length of 6, and a minimum number of peptides of 2.

### MD simulations

For SMYD2-peptide simulations, each peptide substrate consisted of seven amino acids. Simulations were solvated in TIP3P (transferable intermolecular potential with 3 points) water, and sodium chloride ions were used to bring the system to physiological salt. Individual systems were each energy minimized, relaxed in the canonical ensemble, equilibrated to atmospheric pressure, and run without restraint in the canonical ensemble. All GROMACS inputs, topology files, and initial coordinates can be downloaded at https://github.com/BradleyDickson.

### Protein crystallization, data collection, and structure determination

For structure determination, full-length human SMYD2 was expressed, purified, and crystallized as described previously (*25*). Briefly, SMYD2 (10 mg/ml) was incubated with 600 μM SAH and crystallized at 20°C in a solution containing 0.1 M tris (pH 7.5), 20.5% polyethylene glycol (PEG) 3350, and 5% ethanol. Crystals were then crushed to generate seeds for growing SMYD2-peptide complex crystals in a solution containing SMYD2 (1.5 mg/ml), 2 mM GWKLNleSKRG peptide, 600 μM SAH, 0.1 M tris (pH 7.5), 20.5% PEG 3350, and 5.9% ethanol. Crystals suitable for diffraction were cryoprotected in a solution containing 0.1 M tris (pH 7.5), 25% PEG 3350, and 5.0% ethanol and then flash cooled in liquid nitrogen. X-ray diffraction was collected at the Advanced Photon Source at beamline 21-ID-F. Diffraction images were processed and scaled using autoPROC and AIMLESS (*35*, *36*). Crystals belong to a tetragonal space group *P*4_2_ with two molecules per asymmetric unit. The structure was solved by molecular replacement using human SMYD2 (PDB: 5KJK) as a search model. Model building and refinement were carried out in Coot and PHENIX, respectively. The final model was validated by MolProbility (*37*). Structural figures were prepared in PyMOL. Coordinates and structure factors were deposited in the PDB with the accession number 6MON.

## Supplementary Material

http://advances.sciencemag.org/cgi/content/full/4/11/eaav2623/DC1

## SUPPLEMENTARY MATERIALS

Supplementary material for this article is available at http://advances.sciencemag.org/cgi/content/full/4/11/eaav2623/DC1 Fig. S1. G9a, SET7/9, and SMYD2 K-OPL substrate selectivity profiles. Fig. S2. G9a, SET7/9, and SMYD2 enzyme assays. Fig. S3. MS/MS analysis of G9a, SET7/9, and SMYD2 reaction products. Fig. S4. Density map for SMYD2-SAH-GWKLNleSKRG structure and comparison of peptide conformation from previous structures. Fig. S5. In vitro SMYD2 assays with protein substrates. Fig. S6. Liquid chromatography (LC)–MS/MS analysis of recombinant PER2. Table S1. Crystallographic data and refinement statistics. Table S2. LoB scores for the human proteome based off SMYD2 K-OPL selectivity profile. Table S3. Missense mutations predicted to affect SMYD2 lysine methylation signaling. Table S4. Recombinant PER2 peptides identified by LC-MS/MS.
