# Supplementary material for: A functional proteomics platform to reveal the sequence determinants of lysine methyltransferase substrate selectivity
Source: Sci Adv. 2018 Nov 28;4(11):eaav2623. doi: 10.1126/sciadv.aav2623 (PMC6261651; doi:10.1126/sciadv.aav2623)
Supplement: http://advances.sciencemag.org/cgi/content/full/4/11/eaav2623/DC1 [file supp_4_11_eaav2623__index.html]

Science Advances | Science Advances

## Supplementary Materials

**The PDF file includes:**

- Fig. S1. G9a, SET7/9, and SMYD2 K-OPL substrate selectivity profiles.
- Fig. S2. G9a, SET7/9, and SMYD2 enzyme assays.
- Fig. S3. MS/MS analysis of G9a, SET7/9, and SMYD2 reaction products.
- Fig. S4. Density map for SMYD2-SAH-GWKLNleSKRG structure and comparison of peptide conformation from previous structures.
- Fig. S5. In vitro SMYD2 assays with protein substrates.
- Fig. S6. Liquid chromatography (LC)–MS/MS analysis of recombinant PER2.
- Table S1. Crystallographic data and refinement statistics.

Download PDF

**Other Supplementary Material for this manuscript includes the following:**

- Table S2 (Microsoft Excel format). LoB scores for the human proteome based off SMYD2 K-OPL selectivity profile.
- Table S3 (Microsoft Excel format). Missense mutations predicted to affect SMYD2 lysine methylation signaling.
- Table S4 (Microsoft Excel format). Recombinant PER2 peptides identified by LC-MS/MS.

**Files in this Data Supplement:**

- Adobe PDF - aav2623\_SM.pdf
