# Supplementary material for: A functional proteomics platform to reveal the sequence determinants of lysine methyltransferase substrate selectivity
Source: Sci Adv. 2018 Nov 28;4(11):eaav2623. doi: 10.1126/sciadv.aav2623 (PMC6261651; doi:10.1126/sciadv.aav2623)
Supplement: http://advances.sciencemag.org/cgi/content/full/4/11/eaav2623/DC1 [file aav2623_SM.pdf]

Science Advances
AAAS

advances.sciencemag.org/cgi/content/full/4/11/eaav2623/DC1

Supplementary Materials for

A functional proteomics platform to reveal the sequence determinants of lysine methyltransferase substrate selectivity

Evan M. Cornett, Bradley M. Dickson, Krzysztof Krajewski, Nicholas Spellmon, Andrew Umstead, Robert M. Vaughan, Kevin M. Shaw, Philip P. Versluis, Martis W. Cowles, Joseph Brunzelle, Zhe Yang, Irving E. Vega, Zu-Wen Sun, Scott B. Rothbart*

*Corresponding author. Email: scott.rothbart@vai.org

Published 28 November 2018, *Sci. Adv.* **4**, eaav2623 (2018)
DOI: 10.1126/sciadv.aav2623

The PDF file includes:

Fig. S1. G9a, SET7/9, and SMYD2 K-OPL substrate selectivity profiles.
Fig. S2. G9a, SET7/9, and SMYD2 enzyme assays.
Fig. S3. MS/MS analysis of G9a, SET7/9, and SMYD2 reaction products.
Fig. S4. Density map for SMYD2-SAH-GWKLNleSKRG structure and comparison of peptide conformation from previous structures.
Fig. S5. In vitro SMYD2 assays with protein substrates.
Fig. S6. Liquid chromatography (LC)–MS/MS analysis of recombinant PER2.
Table S1. Crystallographic data and refinement statistics.

Other Supplementary Material for this manuscript includes the following:

(available at advances.sciencemag.org/cgi/content/full/4/11/eaav2623/DC1)

Table S2 (Microsoft Excel format). LoB scores for the human proteome based off SMYD2 K-OPL selectivity profile.
Table S3 (Microsoft Excel format). Missense mutations predicted to affect SMYD2 lysine methylation signaling.
Table S4 (Microsoft Excel format). Recombinant PER2 peptides identified by LC-MS/MS.

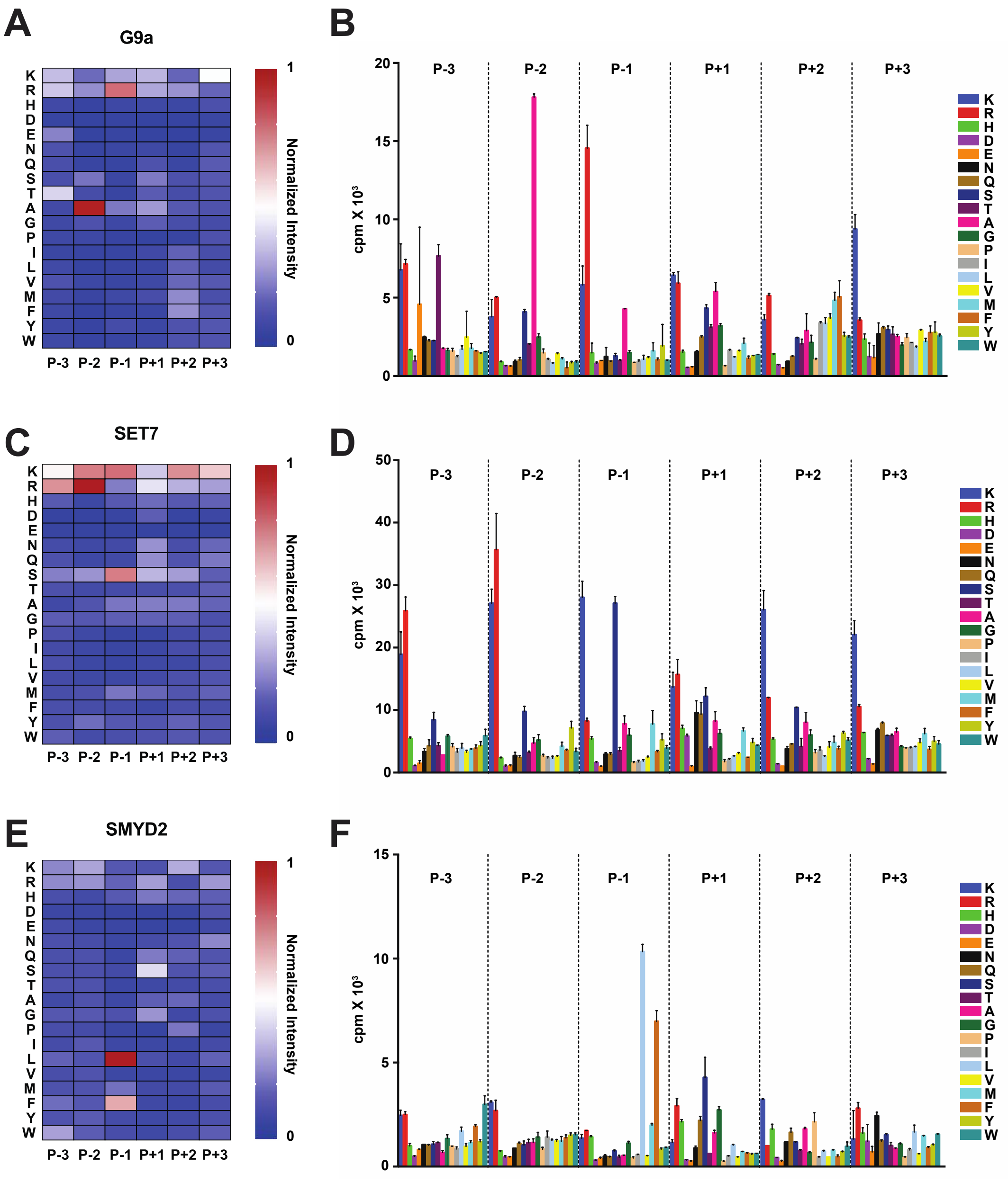

Fig. S1. G9a, SET7/9, and SMYD2 K-OPL substrate selectivity profiles. (A,C,E) K-OPL substrate selectivity profiles for G9a, SET7/9, and SMYD2. Mean results of two independent K-OPL screens for each enzyme are reported as globally-normalized heat maps. The color code is proportional to creation of enzyme product, where red (1) is most active and blue (0) is least active. Rows show the identity of each fixed residue, columns show position within the sequence. (B,D,F) Raw K-OPL data for G9a, SET7/9, and SMYD2. Mean results of two independent K-OPL screens are shown with error bars representing one standard deviation from the mean

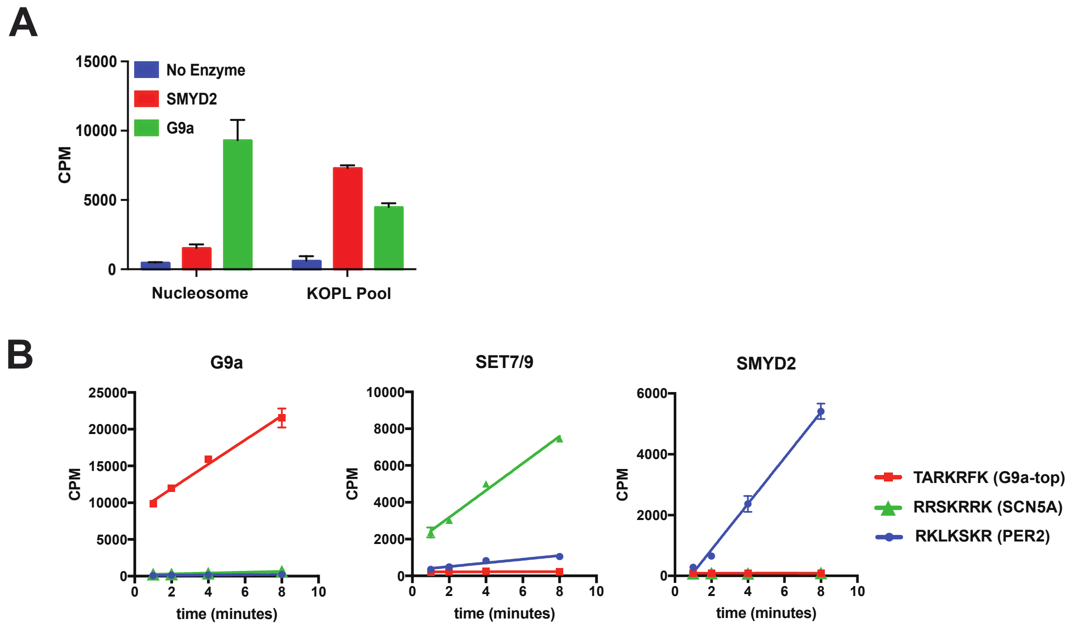

Fig. S2. G9a, SET7/9, and SMYD2 enzyme assays. (A) Comparison of nucleosomes and K-OPL peptides as substrates for SMYD2. Scintillation proximity assay using a pool of all 114 K-OPL sets or a biotinylated human nucleosome (EpiCypher # 16-0006) as substrates. (B) Initial rate measurements of peptides corresponding to the best G9a (TARKRFK, red), SET7/9 (RRSKRRK, green), and SMYD2 (RKLKSKR, blue). Mean of three independent experiments are shown with error bars representing s.e.m.

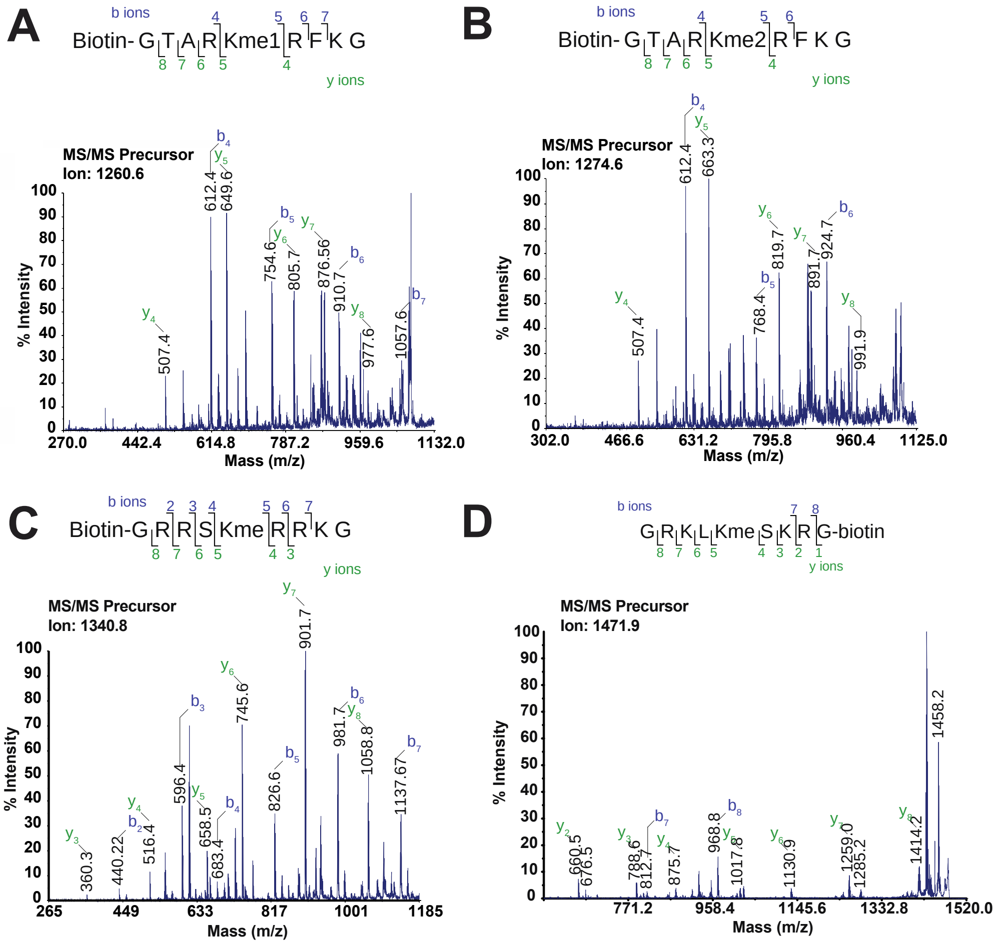

Fig. S3. MS/MS analysis of G9a, SET7/9, and SMYD2 reaction products. (A, B) Tandem mass spectrometry of G9a reactions products from precursor ions 1260.4 (A) or 1274.6 (B). (C) Tandem mass spectrometry of SET7/9 reaction product with precursor ion 1340.8. (D) Tandem mass spectrometry of SMYD2 reaction product with precursor ion 1471.9.

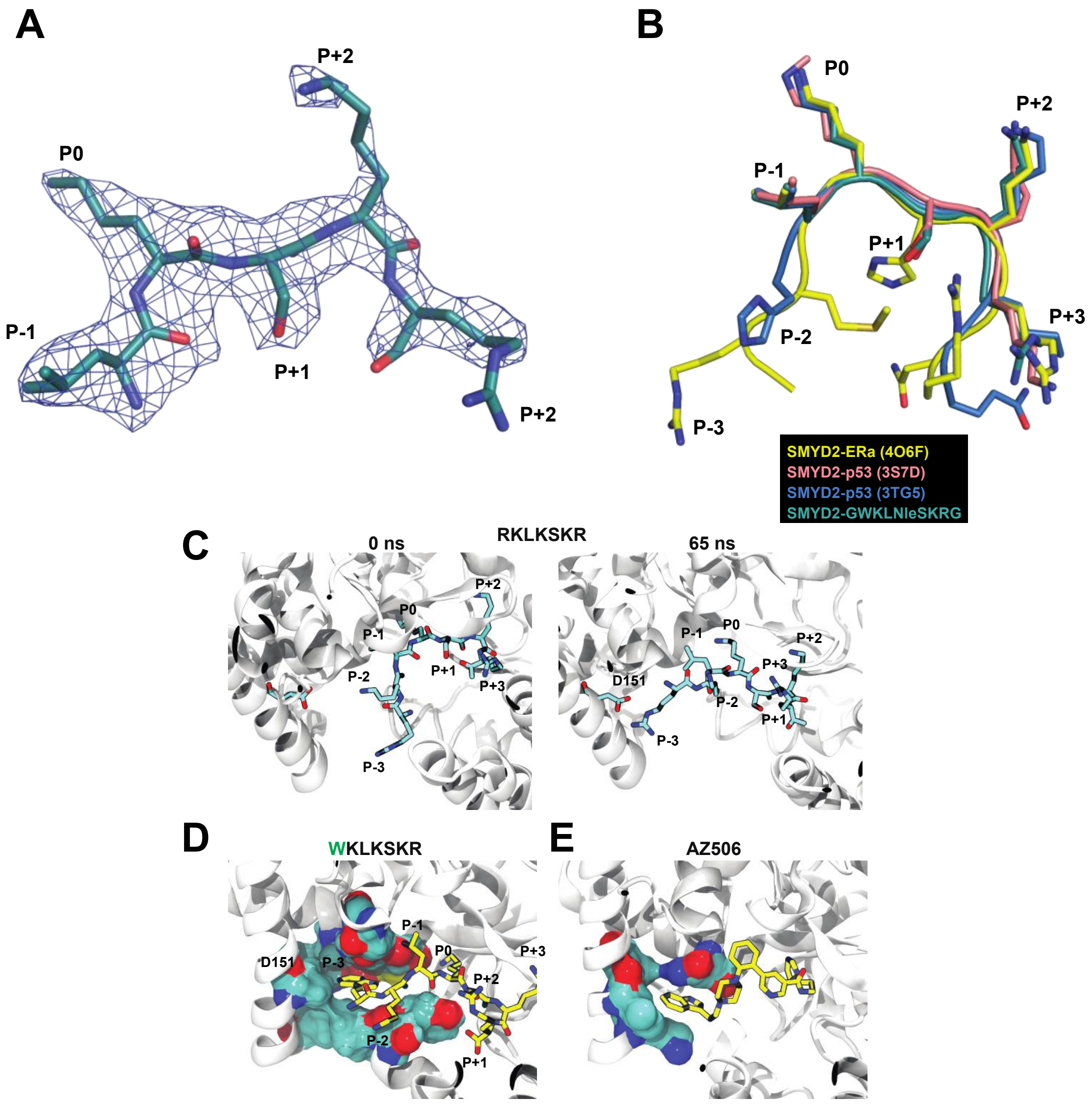

F

	Vmax (c.p.m. x min^{-1})	K_m (µM)
RKLKSKR	5397 ± 789.8	2.68 ± 1.5
DKLKSKR	5014 ± 688.4	2.13 ± 1.4
SHLKSKK	1713 ± 81.9	6.45 ± 0.9
WKLKSKR	1626 ± 124.4	4.92 ± 1.3

Fig. S4. Density map for SMYD2-SAH-GWKLNleSKRG structure and comparison of peptide conformation from previous structures. (A) 2mFo-Fc density map of GWKLNleSKRG peptide from SMYD2-GWK- LKNleSKRG-SAH structure. (B) Structural alignments of peptide substrates from the indicated SMYD2 co-structures. (C) Conformation of RKLKSKR peptide at the beginning (left) and after 65 ns of MD simulation. (D) Conformation of WKLKSKR peptide from the 500 ns pose in MD simulations. (E) Structure of AZ506 bound to SMYD2 (PDB: 5KJN). (F) Kinetic parameters associated with data shown in Figure 3D.

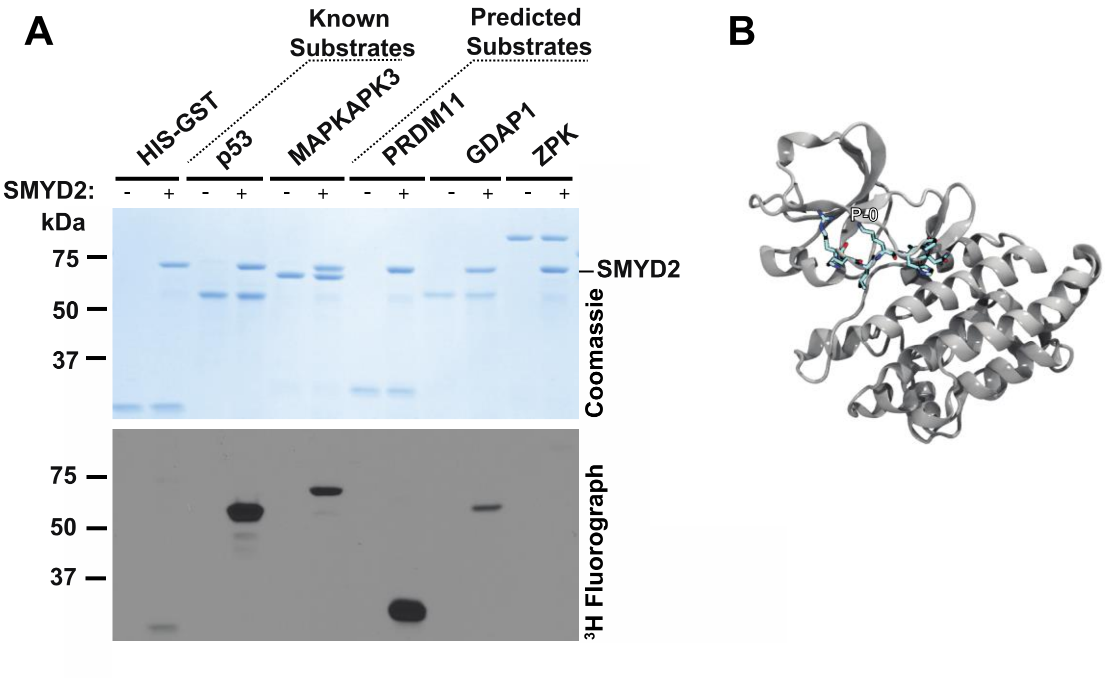

Fig. S5. In vitro SMYD2 assays with protein substrates. (A) Coomassie stained SDS-PAGE of recombinant control (HIS-GST), known substrates (p53, MAPKAPK3), and predicted substrates (PRDM11, GDAP1, MAP3K12) in the absence and presence of SMYD2. Reactions were carried out with 1 μg of SMYD2, 1 μg of substrate, and 1 μCi of 3H-SAM. 3H fluorograph shown below after 24 hour exposure at -80 °C (B) Cartoon repre- sentation of ZPK (PDB: 5CEN) showing plus and minus three amino acids from the target lysine (PO) as stick models.

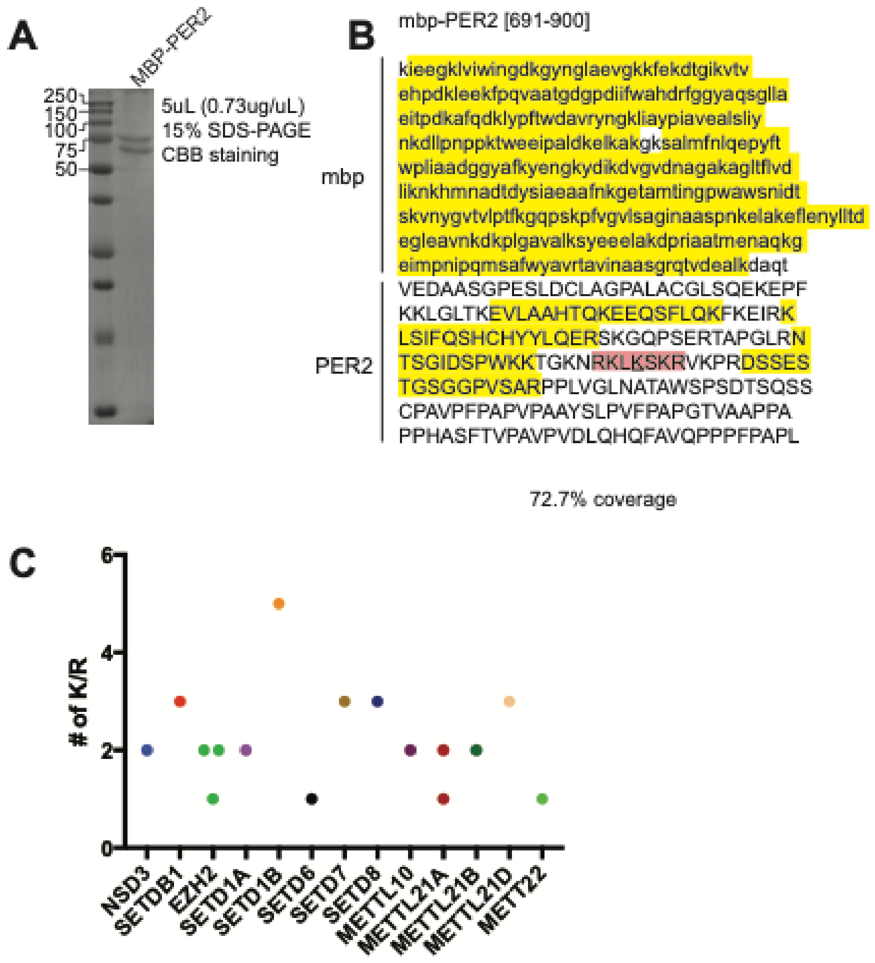

Fig. S6. Liquid chromatography (LC)–MS/MS analysis of recombinant PER2.
(A) Coomassie stained SDS-PAGE of purified MBP tagged PER2. (B) Cumulative sequence coverage is shown in yellow from Asp-N, Trypsin, and Arg-C digested MBP-PER2 following LC-MS-MS analysis. The SMYD2 methylation motif is indicated in red. The peptide sequence corresponding to the maltose binding protein (MBP) tag is in lowercase and PER2 is in uppercase. (C) Analysis of lysine and arginine content for known non-histone KMT substrates. The number of lysine and arginine residues within 3 amino acids of the modified lysine for substrates reported by Biggar et al (7).

Table S1. Crystallographic data and refinement statistics.

**Data**	
Space group	$P4_2$
Cell parameters	
a, b, c (Å)	153.73, 153.73, 53.46
Wavelength (Å)	0.97872
Resolution (Å)	153.73 - 2.71
R_{merge}	0.098 (1.052)
R_{pim}	0.055 (0.795)
$CC_{1/2}$	0.998 (0.519)
Redundancy	4.2 (4.2)
Unique reflections	34429
Completeness (%)	99.9 (100)
⟨I/σ⟩	11.9 (1.4)
**Refinement**	
Resolution (Å)	51.3-2.71 (2.81-2.71)
Molecules/AU	2
R_{work}	0.2105 (0.323)
R_{free}	0.227 (0.341)
Ramachandran plot	
Residues in favored	98.1%
Residues in allowed	1.9%
RMSD	
Bond lengths (Å)	0.006
Bond angels (°)	1.06
No. of atoms	7074
Protein	6906
Peptide	97
Water	7
Zinc	6
SAH	2
Glycerol	1
B-factor (Å^2)	75.7
Protein	75.7
Peptide	84.8
Water	56.9
Zinc	67.7
SAH	59.6
Glycerol	86.1
